# Differential effects of concentric and eccentric contractions on the primary motor cortex in healthy young and elderly participants

**DOI:** 10.3389/fnagi.2025.1553277

**Published:** 2025-06-06

**Authors:** Marion Desachy, Nelly Héraud, Julien Lagarde, Simon Pla, Alain Varray

**Affiliations:** ^1^EuroMov Digital Health in Motion, Univ Montpellier, IMT Mines Ales, Montpellier, France; ^2^Direction Scientifique et Recherche, Groupe Clariane, Lodève, France

**Keywords:** EEG, movement related cortical potential, muscle weakness, aging, eccentric

## Abstract

**Introduction:**

Aging is associated with a decline in musculoskeletal function, particularly muscle weakness, which affects a significant proportion of older adults and is associated with reduced quality of life and increased mortality. Two major contributors to age-related muscle weakness are muscle atrophy and cortical alterations. Eccentric exercise has been identified as a promising intervention to counteract these declines, as it has the potential to increase both muscle mass and cortical activity in young people. However, while the benefits of eccentric contractions on muscle mass in older adults are well documented, their effects on cortical activity, particularly in the lower limbs, remain unclear. The aim of this study was to compare cortical activity during concentric and eccentric quadriceps contractions of young and older adults.

**Methods:**

This prospective study included 32 healthy participants: 17 young (23 ± 4 years, 6 women, 11 mens) and 15 older (62 ± 7 years, 7 women, 8 mens). Muscle strength was assessed using an isokinetic ergometer, muscular activity with electromyography electrodes positioned on quadriceps, and cortical activity using electroencephalography (EEG). Participants performed 40 concentric and 40 eccentric voluntary contractions against 20% of their maximal voluntary isometric contraction. EEG data were processed to analyze motor-related cortical potentials, specifically the negative potential (NP). The NP was divided into two main components: latency and amplitude as indicators of cortical activity during movement preparation and execution.

**Results:**

There were no significant differences in participants characteristics between groups, except for age. Muscular activity was lower during eccentric than concentric contractions (*p* < 0.05). Cortical activity was significantly lower in older compared to young adults, which was reflected in reduced NP latency across several electrodes (Cz, *p* = 0.03; C4, *p* = 0.02; FC2, *p* = 0.02). However, regarding NP amplitude, it was significantly higher during eccentric contractions in Cz, C4, FC5, and C2 electrodes (*p* < 0.05) across both age groups.

**Conclusion:**

This study is the first to investigate cortical activity during eccentric lower limb contractions in older adults. The results suggest that eccentric contractions induce greater cortical activation compared to concentric, even in older adults who generally exhibit reduced cortical activity. These findings support the potential of eccentric as an effective intervention to improve motor function and muscle strength in older adults.

## 1 Background

Aging is a complex and inevitable biological process, associated with the decline of multiple systems such as the cardiovascular, respiratory, musculoskeletal or immune systems, increasing vulnerability to chronic disease and infections (López-Otín et al., 2013). Risk of lower limb muscle weakness is increasing during aging. Defined as the loss of muscle strength, this comorbidity affects between 9 and 13% of individuals over 60 years and more than 50% of those over 80 years (Cruz-Jentoft et al., 2010). It is associated with a reduced quality of life, postural instability and increased risk of falls and injuries, and with an increased hospitalization rate (Orr, 2010). Additionally, a low quadriceps strength is associated with an increased mortality rate (Newman et al., 2006).

In order to determine the most appropriate intervention to increase quadriceps strength when muscle weakness occurs, it is essential to understand its underlying main factors. In literature, two stand out as primary contributors: muscle atrophy and cortical alterations (Clark et al., 2015; Clark and Fielding, 2012; Clark and Taylor, 2011). Muscle atrophy is characterized by alterations in muscle fiber, loss of motor units and impaired protein synthesis (Degens, 2007; Narici et al., 2004; Ryall et al., 2008; Seene and Kaasik, 2012). While several studies, particularly in clinical populations such as individuals with chronic obstructive pulmonary disease (COPD), have estimated that muscle atrophy may account for up to 50–60% of age-related muscle weakness (Bernard et al., 1998; Menon et al., 2012; Seymour et al., 2010), evidence from large longitudinal studies in healthy older adults suggests a much smaller contribution. Delmonico et al. (2009) reported that changes in muscle mass explained only about 6–8% of the variance in strength decline, indicating that other factor play an important role (Delmonico et al., 2009). The cortical alterations are also a significant contributor of muscle weakness since they explain between 20 and 30% of the variance of muscle force (Clark et al., 2015; Clark and Taylor, 2011). Among abnormalities, a decrease in gray and white matter, a reduction in dendrite arborization and spine number, and neural dysfunction are classically reported (Clark and Fielding, 2012). Consequently, to fight efficiently against muscle weakness, and maximize strength improvement, it is thus necessary to promote interventions that target both muscle atrophy (i.e., increasing muscle mass) and cortical alterations (i.e., increasing cortical activity).

Among interventions well documented (among them nutrition, electrostimulation or stretching…), eccentric exercise appears to be a potential promising and relevant candidate. First of all, it is a safe intervention mode already used in elderly. Among its main advantages, this mode of contraction allows work at higher intensities with a lower perception of effort, oxygen consumption, and energy cost than concentric ones (Kim et al., 2022). This implies that a greater amount of work can be easily performed even in conditions with reduced functional capacities as in older individuals. Further, its efficacy on the determinants of muscle strength gain is documented by numerous studies. Eccentric contractions significantly increase quadriceps muscle mass and strength in both young and old people. Studies indicate up to 24% quadriceps hypertrophy and significant strength gains in young individuals following eccentric training (Brasileiro et al., 2011; Higbie et al., 1996; Hortobágyi et al., 1996; Leong et al., 2014). Similar results were observed, with up to 22% increases in quadriceps muscle thickness (Čretnik et al., 2022; Gluchowski et al., 2015; Katsura et al., 2019; LaStayo et al., 2009; Molinari et al., 2019). These studies suggests that eccentric contractions could be efficient to fight against muscle atrophy, the first key factor of age-related muscle weakness. The literature dedicated to eccentric training effects on cortical activity, is unfortunately less conclusive, due to a limited number of studies carried out on this topic. Nonetheless, understanding the cortical mechanisms underlying eccentric training is equally essential. In clinical contexts such as pulmonary rehabilitation, approximately half of COPD patients fail to achieve significant strength gains despite mechanical loading, which may be due to insufficient neural adaptations (Desachy et al., 2023). Identifying whether eccentric contractions promote cortical activation could therefore help optimize rehabilitation strategies by targeting both muscular and neural pathways of adaptation. However, some data are available concerning acute cortical responses during eccentric contractions. In young subjects, the literature highlights a greater cortical activity measured by EEG during the preparation and execution phases of the movement (Koohestani et al., 2020). In older people, fMRI indicate that a greater number of cortical regions are activated during eccentric movement (Yao et al., 2014). Taken together, these studies are consistent with the interest of eccentric contraction increase of cortical circuit functioning.

Unfortunately, findings on older people were systematically obtained on the upper limb. Therefore, these results cannot be directly extrapolated to the lower limbs due to their association with functional independence (e.g., gait, walking speed, fall prevention and more generally autonomy). This issue is all the more serious as some elements can act to induce different results in lower limbs. Indeed, researches have demonstrated that movements of the upper and lower limbs are associated with different patterns of cortical activation. Specifically, upper limb movements are characterized by greater cortical lateralization measured by fMRI, with substantial excitatory input to the contralateral motor cortex and significant interhemispheric inhibition, reflecting their role in fine and controlled movements (Volz et al., 2015). In contrast, lower limb movements involve more central, symmetrical interactions between hemispheres with reduced inhibition (Harirchian et al., 2008, fMRI study). This distinction is related to the nature of the tasks: lower limb movements are generally less analytic and more coordinated (Martínez-Expósito et al., 2017). Consequently, these movements generate less intense cortical activity compared to upper limb movements, which require higher activity due to the complexity and fine motor control involved (Wheaton et al., 2008). Furthermore, the literature reports systematically lower cortical activity measured by EEG during movements of the lower limbs (potentially up to 50% lower compared to similar movements of the upper limbs) (Kline et al., 2016; Luft et al., 2002). Consequently, it remains uncertain whether eccentric movements of the lower limbs generate sufficient cortical activity to effectively combat age-related declines in both age-related brain alterations and muscle weakness.

In summary, eccentric exercise appears promising because it induces positive adaptations on major key factors of muscle weakness (atrophy and cortical alterations) in young subjects. However, no strong knowledge is available for older adults during lower limb eccentric contractions. Before proposing its use in a pathological context, we need to determine its effects on healthy older adults and on the lower limbs. Thus, the objective of this study was to compare cortical activity during quadriceps concentric and eccentric contractions in young vs. older healthy participants. The inclusion of a young adult group was intended to validate the experimental protocol in a population with preserved neuromuscular function. This ensured that the protocol was able to detect expected cortical activation patterns during lower limb contractions, before evaluating potential alterations related to aging.

## 2 Materials and methods

### 2.1 Participants

The prospective study included a total of 38 healthy participants. To be included, participants had to be right leg dominant and aged between 20 and 35 years for the young group, or between 50 and 70 years for the older group. The older group was composed of functionally healthy individuals without clinically significant muscle weakness. This choice allowed us to isolate age-related cortical effects independently of pathological conditions such as sarcopenia, and to establish a clear baseline for future comparisons.

The exclusion criteria were the existence of a lower limb trauma during the last 6 months, the inability to perform full knee extensions, a chronic illness, a neuromuscular pathology, a possession of any medical device (pacemaker, etc.) and the use of treatment known to modify neuronal activity [antidepressants, excessive consumption of caffeine/theine, i.e., more than 5 doses per day (Nehlig et al., 1992)]. Finally, 32 subjects were analyzed (6 subjects were excluded for the poor signal quality) and assigned to young (17 participants, mean age 23.5 ± 4.2 years, 6 women, 11 mens) or older group (15 participants, mean age 61.9 ± 5.7 years, 7 women, 8 mens). All included individuals signed informed written consent before participating to the study. In accordance to the Declaration of Helsinki, the study was approved by the local Ethics Committee (Institutional Review Board, permit number 2101B).

### 2.2 Procedures

The following procedures were based on the protocol described by Fang et al. (2001, 2004).

#### 2.2.1 Dominant leg identification

The subject’s dominant leg was determined based on three distinct tests: (1) ascending a step, (2) striking a ball with foot, and (3) experiencing forward imbalance. The leg naturally used for at least 2 out of the 3 tests was considered as the dominant leg (de Ruiter et al., 2010). Only participants with a right dominant leg were included in the study, for standardization purposes and due to equipment setup constraints.

#### 2.2.2 Strength assessment

An isokinetic ergometer, the BIODEX 3 (Biodex, Shirley, New York), was used to assess the muscle strength of the dominant quadriceps of the subjects.

Participants were comfortably seated on the BIODEX 3 isokinetic ergometer. Adjustments were made to ensure hip and knee angles of 90°. The lever arm was properly adjusted to ensure attachment of the arm in line with the subject’s kneecap and 2 cm above the ankle. The subject’s right (dominant) leg was attached to the ergometer. The subject’s shoulders, torso and arms were secured with belts so that only movements at the knee joint were possible.

Angle and force torque signals were recorded using the DELSYS system (Trigno*™* Wireless Systems, Delsys Inc., United States), at an acquisition frequency of 518 Hz.

After a standardized warm-up consisting of concentric and eccentric contractions performed at 120°/s, 70°/s, and 30°/s, the maximal voluntary isometric contraction (MVIC) of the quadriceps was assessed. For this, the leg was positioned at 90° and the ergometer was locked in an isometric position. The subjects were instructed to develop maximal force for 5 s, as if they were attempting to extend the leg (Babault et al., 2001). The contraction was considered valid if a plateau was maintained for at least 2 s. Three maximal contractions were performed, each separated by 1 min of passive rest. If the variation among the three contractions exceeded 10%, a fourth contraction was conducted to ensure three reproducible contractions. Based on the average MVIC value obtained, an external load (kg) corresponding to 20% of the MVIC was calculated and physically attached to the participant’s ankle (Fang et al., 2001).

Voluntary concentric and eccentric contractions of the quadriceps were performed against this fixed load (20% of MVIC). Concentric contractions involved raising the leg from 100° to 165° (i.e., from vertical to horizontal position), while eccentric contractions involved lowering the leg from 165° to 90°. Each movement was executed over 3 s, corresponding to a controlled angular velocity of 22°/s (Babault et al., 2001). Although the setup involved an isokinetic dynamometer, the fixed nature of the resistance made the task functionally isotonic.

#### 2.2.3 Electroencephalography recording

The Starstim 20 system (Neuroelectrics, Barcelona, Spain) was used to record EEG signals from the scalp.

**FIGURE 1:**
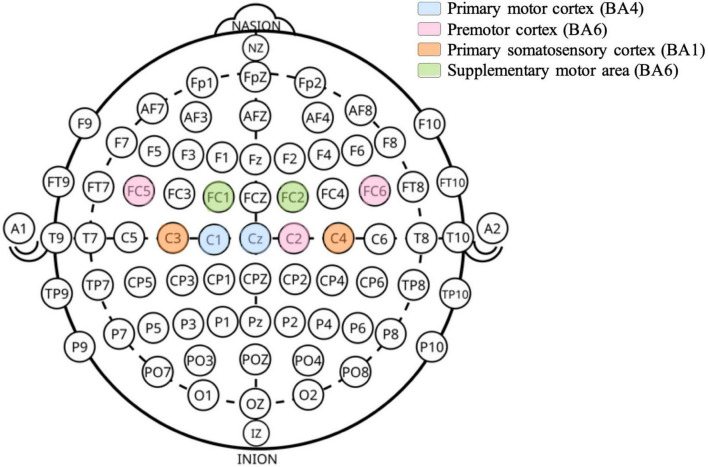
Scalp positions of EEG electrodes used. Electrode labels follow the international 10–20 system. The underlying cortical areas and their associated functions are indicated based on anatomical mapping studies (Scrivener and Reader, 2022).

The EEG cap containing the electrodes (NG Geltrode, Neuroelectrics) was positioned on the participant’s head with Cz at the center of the head. The electrode positioning was based on the International 10–20 System (Klem et al., 1999). In this experimental design, 9 electrodes (Cz, C1, C2, C3, C4, FC1, FC2, FC5, FC6) were used and positioned overlying the sensorimotor areas of ipsi- and contra-lateral hemispheres (Figure 1). Cz and C3 were considered as the main electrodes due to their proximity to the leg motor cortex area.

Conductive gel (Signa Gel^®^) was injected into each electrode using a syringe. A color-coded map indicating electrode impedance was displayed to improve the connection between the electrodes and the scalp. Elevated impedance was typically reduced by applying more gel or gently pressing the electrode, in order to keep impedance of each electrode below 10 kΩ. References electrodes (CMS, DRL) were placed over the right earlobe using an ear clip. The EEG was sampled at 500 Hz. The EEG device was connected using WIFI and controlled by the computer through a software interface (Neuroelectrics Instrument Controller, NIC v 2.0).

#### 2.2.4 Electromyography recording

The DELSYS wireless dynamic EMG system (TrignoTM Wireless Systems, Delsys Inc., United States) was used to record the electrical activity of the vastus lateralis, vastus medialis, and rectus femoris of the dominant leg. Before each electrode placement, the skin was shaved, abraded, and cleaned with alcohol. EMG signal quality was automatically assessed by the Delsys Trigno system before each recording using the built-in diagnostic function. This tool provides a visual indicator (green/orange/red) for each sensor, reflecting the quality of electrode-skin contact. We verified that all sensors displayed green status prior to data collection, which corresponds to low contact resistance and optimal signal acquisition (typically equivalent to impedance values below ∼5 kΩ). This ensured consistent EMG signal quality across participants. The sampling frequency was 2148 Hz.

The wireless EMG signals were transmitted to the EMG acquisition software via the electrodes pasted on muscle surfaces, and then processed and analyzed using the EMG software (EMGworks Analysis and MATLAB 2021b).

### 2.3 Protocol

After the participants’ arrival, the dominant leg was identified. The EMG electrodes were attached on the dominant leg, the EEG cap and electrodes were placed and the impedance checked. Once equipped, the participant takes a seat on the ergometer and adjustments were made.

Subjects performed a warm-up composed of 3 sets of 10 concentric-eccentric contractions (leg extension-flexion) at 120°/s, 70°/s, and then 30°/s. After that, the MVIC was assessed. To familiarize with contractions, subjects performed 10 concentric and 10 eccentric contractions to well understand to movement and respect the timing of the task. After that, subjects began with either concentric or eccentric contractions (randomly). They performed 40 concentric contractions (2 sets of 20) and 40 eccentric contractions (2 sets of 20) against a load equal to 20% of their MVIC. Each contraction was preceded by a 3-s isometric contraction (Fang et al., 2001). The movement was calibrated using vocal instructions (“start”, “go”, “stop”). There was a 20-s rest between each contraction, 5 min of rest between the two sets, and 10 min of rest between the two modes of contractions.

During the contractions, subjects were instructed to remain as relaxed as possible, without moving, speaking, and to focus on a visual cue in front of them (a black cross (approximately 10 × 10 cm), drawn with a marker on a whiteboard, was placed at eye level about 1.5 meters in front of the participant).

### 2.4 Data analysis

#### 2.4.1 EMG pre-processing

To preprocess the EMG signal, a Butterworth high-pass filter with a cutoff frequency of 10 Hz was applied to remove movement artifacts, followed by a low-pass filter with a cutoff frequency of 400 Hz to eliminate high-frequency noise. The amount of muscle electrical activity was expressed as the root mean square (RMS) of the EMG recording.

#### 2.4.2 EEG pre-processing

**FIGURE 2:**
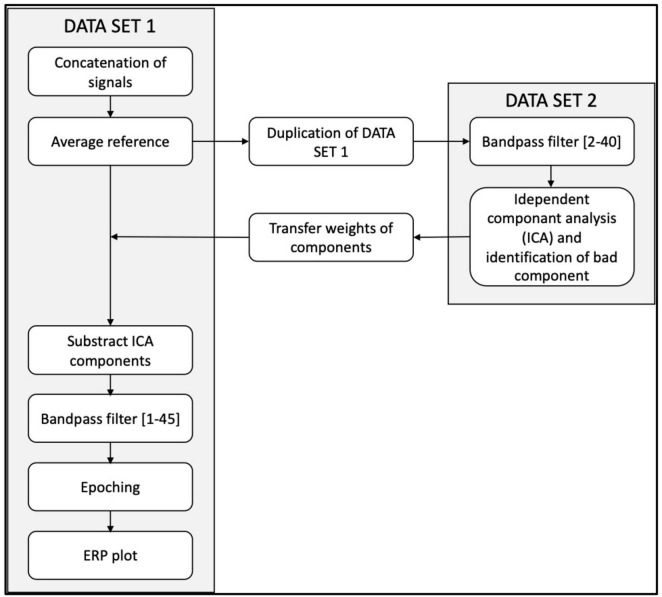
Pre-processing EEG pipeline.

Off-line signal processing and analyses were performed using EEGLAB open-source toolbox version 2022.0 (Delorme and Makeig, 2004). Raw continuous EEG data were preprocessed using the pipeline describe in Figure 2. First bandpass filtered between 1 and 40 Hz. To reduce high-amplitude, non-stationary artifacts prior to ICA decomposition, we applied Artifact Subspace Reconstruction (ASR) with a burst criterion of 20 and a window criterion of 0.25. This method allowed automatic detection and correction of transient artifacts by comparing signal characteristics to a baseline estimated from clean EEG segments. ICA was then performed using the Infomax algorithm (runica function in EEGLAB). Artifactual components (e.g., ocular, muscle) were identified using ICLabel and removed. A visual inspection of the cleaned signal was conducted afterward to ensure artifact-free epochs.

Data were segmented into 10-s windows time-locked to each trigger (from –5 to +5 s). Only participants with at least 10 artifact-free trials per condition (eccentric and concentric) were included in the final analysis to ensure quality (Borràs et al., 2022; Boudewyn et al., 2018).

#### 2.4.3 Motor related cortical potential

MRCPs were selected as the primary cortical marker to replicate and extend the work of Fang et al. (2001), who used this approach to compare cortical activation during concentric and eccentric contractions (Fang et al., 2001). MRCPs are well-established, temporally precise indicators of voluntary movement preparation and execution, and allow for trial averaging and robust inter-group comparisons.

For each EEG channel, MRCPs were computed by averaging across artifact-free trials for each condition. EEG epochs were baseline-corrected using a pre-movement resting segment (-10 s), and the mean baseline value was set as the y-axis reference. A linear regression was applied to the descending slope of the waveform leading to the negative peak (NP). The onset of cortical motor preparation was defined as the intersection between this regression line and the baseline.

**FIGURE 3:**
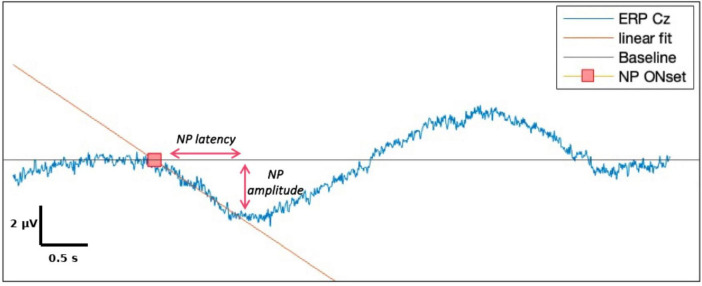
Components of negative potential (NP) and illustrations of their measurements.

NP latency was defined as the time interval between this onset point and the most negative value (NP). NP amplitude was calculated as the difference between the baseline level and the NP (Figure 3). All waveforms were visually inspected, and manual corrections were made when needed to ensure reliability (Shibasaki and Hallett, 2006).

### 2.5 Statistical analysis

All statistical analyses were performed using JASP software [JASP Team (2022). JASP (Version 0.16.3)].

Before comparing the participant’s characteristics, we assessed the normality of the data distribution using the Shapiro-Wilk test. Since all variables met the assumption of normality (*p* > 0.05 for age, body weight, height, BMI and quadriceps strength), parametric tests were used. An unpaired *t*-test was used to compare differences between young and older participants. A chi-square (χ^2^) test was used to compare the distribution of sex (male vs. female) between the two age groups.

For all analyses, data were first averaged across valid trials for each participant and condition. Statistical tests were then conducted on these participant-level averages for EMG RMS values at each muscle, and MRCP features at each EEG electrode.

To compare MRCPs between concentric and eccentric contractions in each group, a two-way repeated-measures ANOVA was performed, with Age Group (young vs. older) as the between-subject factor and Contraction Mode (concentric vs. eccentric) as the within-subject factor. Assumptions of normality (Shapiro–Wilk test) and homogeneity of variances (Levene’s test) were confirmed for all dependent variables (*p* > 0.05). The assumption of sphericity for the within-subject factor was assessed using Mauchly’s test.

The effect size was estimated with Partial Eta Squared ($\eta_{p}^{2}$). A Partial Eta Squared of 0.01, 0.06 or 0.14 indicated, respectively, small, medium or large effect.

All results are reported as mean ± standard deviation (SD). An alpha threshold of 0.05 was considered significant for all statistical tests.

## 3 Results

### 3.1 Participants characteristics

Participant’s characteristics are given in Table 1. Except for age, the participant’s characteristics were non-significantly different between groups.

**TABLE 1:** Participant’s characteristics.

Variable	Young group (*n* = 17)	Older group (*n* = 15)	*P*-value
Age (years)	23.5 ±4.2	61.9 ± 5.7	<0.001
Male/female	12/5	8/7	ns (0.31)*
Body weight (kg)	63.5 ± 8.6	67.6 ±13.6	ns (0.33)
Height (cm)	168.1 ±10.5	169.2 ±7.1	ns (0.71)
BMI	22.2 ±2.9	24.1 ±3.1	ns (0.09)
Q_MVIC_ (N.m)	196.9 ±60.7	162.6 ±41.4	ns (0.08)
Q_MVIC_ (kg)	50.2 ±13.4	42.5 ±11.2	ns (0.08)

BMI, body mass index; Q_MVIC_, quadriceps maximal voluntary isometric contraction. ns, non-significant statistical difference. (Independent sample *t*-test were used for all variables except for the comparison of male and female, a Chi-squared was used).

*Means that a Chi-squared test was used.

### 3.2 Muscular activity

The ANOVA conducted on the RMS values of the EMG signals from the rectus femoris, vastus lateralis, and vastus medialis, revealed no significant interactions between age group (young vs. older) and contraction mode (concentric vs. eccentric) across the three muscles (*p*-values related to F_contraction*age_ ranging from 0.22 to 0.49).

Additionally, there was no significant effect of age, indicating that EMG activity did not differ significantly between younger and older participants (*p*-values related to F_age_ ranging from 0.14 to 0.74).

However, a significant effect of contraction mode was found for both the rectus femoris and vastus lateralis, with a significantly lower RMS values observed during eccentric contractions compared to concentric ones (respectively, *F* = 8.86, *p* < 0.01, $\eta_{p}^{2}$ = 0.22 and *F* = 6.65, *p* = 0.01, $\eta_{p}^{2}$ = 0.17). No significant effect of contraction mode was found for the vastus medialis (*F* = 1.66, *p* = 0.21, $\eta_{p}^{2}$ = 0.05).

### 3.3 Cortical activity

#### 3.3.1 NP Amplitude (μV)

**FIGURE 4:**
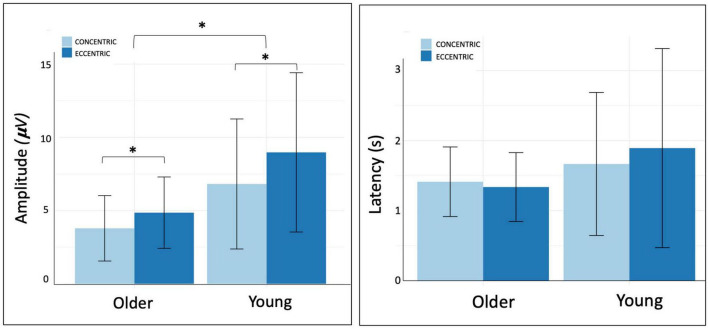
Amplitude and latency of negative peak on Cz electrode.

NP amplitude on Cz, C2, C4, FC5 were significantly higher during eccentric contractions compared to concentric ones (respectively, *p* = 0.02, $\eta_{p}^{2}$ = 0.17; *p* < 0.001, $\eta_{p}^{2}$ = 0.39; *p* = 0.005, $\eta_{p}^{2}$ = 0.27; *p* = 0.01; $\eta_{p}^{2}$ = 0.21) (Table 2 and Figure 4). FC1 amplitude revealed a significant interaction between the age group and the contraction mode (*p* = 0.04, $\eta_{p}^{2}$ = 0.19). Older adults exhibited reduced cortical activity during concentric contractions and greater activity during eccentric contractions, whereas younger participants showed the opposite pattern, with higher cortical activity during concentric compared to eccentric contractions.

**TABLE 2:** ANOVA results on NP amplitudes (in μV) on age and contraction mode factors.

		Means SD		ANOVA results				
Area	Modes	Older	Young		Contraction * age	Contraction mode (concentric vs. eccentric)	Group age (young vs. older)
C1	Concentric Eccentric	2.39 ± 1.63 2.80 ± 1.80	2.96 ± 1.58 2.41 ± 1.03	**F** **p** $\eta_{p}^{2}$	2.60 0.12 0.08	0.06 0.81 <0.01	0.04 0.84 <0.01
C2	Concentric Eccentric	1.86 ± 1.24 3.19 ± 2.16	2.21 ± 0.95 3.06 ± 1.21	**F** **p** $\eta_{p}^{2}$	0.75 0.40 0.03	15.79 ***<0.001*** 0.39	0.05 0.82 <0.01
C3	Concentric Eccentric	2.55 ± 1.34 2.91 ± 1.52	3.36 ± 1.81 2.92 ± 1.25	**F** **p** $\eta_{p}^{2}$	1.83 0.19 0.06	0.02 0.89 <0.01	0.86 0.36 0.03
C4	Concentric Eccentric	2.21 ± 1.24 3.44 ± 2.06	2.78 ± 1.03 3.38 ± 1.24	**F** **p** $\eta_{p}^{2}$	1.15 0.29 0.04	9.66 ***0.005*** 0.27	0.31 0.58 0.01
Cz	Concentric Eccentric	3.86 ± 2.21 5.92 ± 3.91	6.75 ± 4.50 8.39 ± 4.96	**F** **p** $\eta_{p}^{2}$	0.07 0.79 <0.01	6.15 ***0.02*** 0.17	4.66 ***0.04*** 0.14
FC1	Concentric Eccentric	2.56 ± 1.11 3.00 ± 1.68	3.22 ± 1.85 2.70 ± 1.05	**F** **p** $\eta_{p}^{2}$	2.41 0.13 0.07	4.79 **0.04** 0.19	0.19 0.67 <0.01
FC2	Concentric Eccentric	2.99 ± 1.62 3.32 ± 1.94	3.00 ± 1.40 3.40 ± 1.53	**F** **p** $\eta_{p}^{2}$	0.01 0.94 <0.01	1.05 0.32 0.04	0.01 0.92 <0.01
FC5	Concentric Eccentric	4.16 ± 1.83 5.60 ± 2.44	4.28 ± 1.60 5.90 ± 2.67	**F** **p** $\eta_{p}^{2}$	0.02 0.88 <0.01	6.80 ***0.01*** 0.21	0.012 0.73 <0.01
FC6	Concentric Eccentric	5.14 ± 2.08 5.35 ± 1.54	5.52 ± 1.79 5.52 ± 2.36	**F** **p** $\eta_{p}^{2}$	0.07 0.79 <0.01	0.08 0.79 <0.01	0.17 0.69 <0.01

Bold and italic values indicate statistically significant results.

Cz NP amplitude was significantly lower in the older group compared to young group whatever the contraction mode (*p* = 0.04, $\eta_{p}^{2}$ = 0.14).

#### 3.3.2 NP Latency (ms)

The analyses revealed a non-significant interaction between contraction mode and age group. Regarding group effect, NP latency on FC2 was systematically lower in the older group whatever contraction mode (*p* = 0.02, $\eta_{p}^{2}$ = 0.16). There were no significant differences on others electrodes (Table 3). No significant effect of contraction mode was observed.

**TABLE 3:** ANOVA results on NP latency (in ms) on age and contraction mode factors.

		Means SD		ANOVA results			
Area	Modes	Older	Young		Contraction * age	Contraction mode (concentric vs. eccentric)	Group age (young vs. older)
C1	Concentric Eccentric	1.29 ± 0.75 1.36 ± 0.68	1.73 ± 1.03 1.43 ± 0.66	F p $\eta_{p}^{2}$	0.80 0.38 0.03	0.31 0.58 0.01	1.69 0.20 0.05
C2	Concentric Eccentric	1.18 ± 0.38 1.44 ± 0.83	1.42 ± 0.71 1.56 ± 0.72	F p $\eta_{p}^{2}$	0.11 0.74 <0.01	1.36 0.25 0.04	1.06 0.31 0.03
C3	Concentric Eccentric	1.41 ± 0.88 1.45 ± 0.67	1.59 ± 0.83 1.36 ± 0.57	F p $\eta_{p}^{2}$	0.41 0.53 0.01	0.20 0.66 <0.01	0.08 0.78 <0.01
C4	Concentric Eccentric	1.67 ± 0.82 1.92 ± 1.23	2.13 ± 1.13 1.81 ± 1.51	F p $\eta_{p}^{2}$	1.18 0.29 0.04	0.02 0.89 <0.01	0.23 0.64 <0.01
Cz	Concentric Eccentric	1.44 ± 0.50 1.41 ± 0.33	1.55 ± 0.64 1.30 ± 0.50	F p $\eta_{p}^{2}$	0.94 0.34 0.03	1.48 0.24 0.05	<0.01 0.97 <0.01
FC1	Concentric Eccentric	1.54 ± 0.87 1.28 ± 0.58	1.69 ± 1.22 1.31 ± 0.63	F p $\eta_{p}^{2}$	0.08 0.79 <0.01	2.04 0.16 0.07	0.19 0.67 <0.01
FC2	Concentric Eccentric	1.31 ± 0.52 1.37 ± 0.56	1.82 ± 0.80 1.89 ± 1.11	F p $\eta_{p}^{2}$	<0.01 0.98 <0.01	0.14 0.71 <0.01	5.88 ***0.02*** 0.16
FC5	Concentric Eccentric	1.45 ± 0.50 1.51 ± 0.80	1.40 ± 0.69 1.41 ± 0.77	F p $\eta_{p}^{2}$	0.02 0.90 <0.01	0.05 0.83 <0.01	0.16 0.69 <0.01
FC6	Concentric Eccentric	1.38 ± 0.52 1.50 ± 0.68	1.35 ± 0.46 1.68 ± 0.71	F p $\eta_{p}^{2}$	0.65 0.43 0.02	3.00 0.09 0.09	0.18 0.67 <0.01

## 4 Discussion

The aim of the study was to compare cortical activity during quadriceps concentric and eccentric contractions in young and older subjects. The major findings were that even though the older group exhibited lower cortical activity compared to the younger group (on Cz and FC2), eccentric contractions were associated higher cortical activity (on Cz, C2, C4, and FC5) in both groups compared to concentric ones. Additionally, a lower muscular activity was found during eccentric contractions compared to concentric ones in young and older group.

From a fundamental perspective, the lower muscular activity observed during eccentric contractions compared to concentric ones aligns with previous research. Eccentric contractions are known to generate high force with reduced neural drive, involving lower motor unit recruitment and firing rates than concentric contractions (Babault et al., 2001; Moritani et al., 1987). This reduced activation is partly due to mechanisms unique to eccentric actions, such as passive force contributions from elastic elements, fewer motor units recruited and the efficient detachment of cross-bridges during lengthening (Kellis and Baltzopoulos, 1998). Additionally, eccentric contractions are metabolically efficient, requiring significantly less ATP and energy expenditure (Enoka, 1996; LaStayo et al., 2003), which makes them advantageous for producing high force levels with minimal muscle activation. This efficiency is reflected in lower EMG amplitudes, indicating that eccentric contractions allow for economical tension development, which is especially useful for strength training and rehabilitation contexts.

Regarding cortical activity, this study revealed two significant differences between age groups. First, elderly individuals showed lower cortical amplitudes at Cz compared to younger participants, regardless of the contraction mode. This reduced amplitude likely reflects decreased cortical motor preparation, as MRCP amplitude, and particularly the negative potential (NP), is known to be a marker of the intensity of cortical recruitment during voluntary movement planning (Shibasaki and Hallett, 2006). This observation aligns with established literature, which attributes such differences to age-related functional and structural cerebral changes. Indeed, numerous studies have highlighted cerebral differences between young and elderly individuals. Structural changes include global cerebral volume reduction, decreased gray matter volume, and lower quality and quantity of white matter (Salat et al., 2009; Seidler et al., 2010). At a functional level, the observed decrease in corticospinal excitability with aging (Fujiyama et al., 2012; Spedden et al., 2018) could be linked to a reduced capacity of elderly individuals to regulate inhibitory processes (Fujiyama et al., 2012). Furthermore, older adults exhibit reduced corticospinal responses during submaximal contractions (Škarabot et al., 2019). In addition, a significantly shorter MRCP onset latency was observed at FC2 in older adults. While this may seem unexpected, FC2 corresponds to the right premotor cortex, which is primarily involved in motor planning. This earlier activation may reflect a compensatory anticipatory mechanism, aimed at maintaining performance despite age-related decline, as suggested by previous findings (Heuninckx et al., 2005; Mattay et al., 2002). More generally, while MRCP amplitude clearly discriminated between groups and conditions, latency appeared less sensitive. This may be due to its tighter temporal constraint within the task and greater interindividual variability, making it less reliable for detecting systematic differences at the group level.

Secondly, this research highlighted greater cortical activity on Cz, C2, C4, and FC5 electrodes during eccentric contractions. These findings are consistent with the literature and confirms several key aspects previously established for upper limb movements or younger individuals. Our results show that the pattern of greater cortical activation during eccentric contractions, compared to concentric contractions, is also observed for the lower limbs (Kline et al., 2016; Luft et al., 2002). Moreover, this pattern persists with aging with a higher cortical activity in eccentric. Functionally, this increased amplitude of the NP suggests that eccentric contractions demand stronger preparatory engagement from the motor system, possibly due to the higher complexity of motor control during lengthening contractions, which require precise modulation of descending commands, and greater reliance on sensorimotor integration (Fang et al., 2001; Enoka, 1996). Additionally, eccentric actions involve controlling an externally imposed movement, rather than generating one from scratch, which has been associated with higher inhibitory demands and the need for fine motor coordination. This may explain the greater activation observed in motor and premotor regions. Another contributing factor may be the relative unfamiliarity of eccentric contractions in daily life. Because these actions are less practiced, they may require more attentional resources and anticipatory planning, further amplifying preparatory cortical activity. This indicates that distinct neuronal control strategies for eccentric movements are maintained whatever the limb engaged and preserved with aging. While these higher cortical activity in eccentric align with the existing literature, our study also revealed an unexpected spatial pattern of cortical activation.

The increased cortical activation during eccentric contractions observed in this study was more localized to central regions (notably Cz, C2, C4, and FC5 electrodes) rather than widespread across the cortex like for upper limb. Although this spatial pattern is consistent with the organization of motor control for lower limb movements, it remains somewhat surprising in light of the literature on eccentric contractions, which has consistently reported broader and more diffuse cortical activation across the cortex during eccentric tasks (Fang et al., 2004). This pattern was consistent across both age groups, contrasting with previous studies reporting more diffuse cortical activation in older adults during eccentric contractions (Fang et al., 2004; Inuggi et al., 2011; Mattay et al., 2002; Perrey, 2018; Sailer et al., 2000; Yao et al., 2014). One relevant explanation lies in the differences between upper and lower limb neural control. Lower limb movements, predominantly engage localized regions in the primary motor cortex, as opposed to the more lateralized and diffuse activation patterns observed for upper limb movements (Volz et al., 2015). These factors likely explain the localized overactivation observed during eccentric contractions in this study, highlighting distinct neural control strategies for lower limb motor tasks.

Finally, these results, and more specifically the higher cortical activity during eccentric contractions, open up very interesting clinical perspectives. Clarifying the cortical effects of eccentric exercise is therefore essential not only to understand the neurophysiological basis of strength gains, but also to improve clinical outcomes by guiding more personalized and targeted rehabilitation interventions, such as with COPD patients. In young individuals, eccentric exercise not only leads to acute cortical response but also induces significant chronic cortical adaptations as an increased cortical activity, a reduction of intracortical inhibition and silent period, as well as an enhancement of corticospinal excitability (Kidgell et al., 2015). In this context, acute responses refer to immediate neurophysiological changes that occur during or shortly after a single session of exercise. In contrast, chronic responses correspond to longer-term adaptations resulting from repeated exposure to exercise over several weeks, such as structural or functional changes. These chronic adaptations are of prime importance since cortical activity is significantly related to the increase of muscle strength after training (Kidgell and Pearce, 2010). In support of this, we found that MRCP amplitude at Cz and C3 was positively correlated with maximal voluntary force. Specifically, Cz amplitude during concentric contractions explained 39% of the variance in strength (*r* = 0.624, *p* < 0.001), whereas Cz and C3 amplitudes during eccentric contractions explained 18% (*r* = 0.421, *p* = 0.017) and 17% (*r* = 0.416, *p* = 0.018), respectively. One could hypothesize that this difference may, at least in part, reflect the greater familiarity and consistency of concentric contractions, which could reduce interindividual variability and strengthen the association with cortical activation. However, this remains a hypothesis and should be further explored in future studies. These correlations suggests that promoting cortical activation may meaningfully contribute to improving strength. Given that our study shows similar acute cortical responses in older adults and in the lower limb, it is reasonable to infer that similar chronic adaptations could be achieved in the elderly. Although this remain to be confirmed by specific studies, there is indirect evidence to support the idea that chronic brain adaptations may be preserved with age, which could play a crucial role in improving muscle strength through this neural mechanism. Indeed, a recent study examining muscular adaptation after 12 weeks of eccentric training in older adults with COPD reported a significant increase in muscle strength without changes in muscle mass (MacMillan et al., 2017). The authors hypothesized that this improvement may stem from cortical adaptations, supporting the potential of eccentric training to induce central nervous system benefits even in clinical populations. Eccentric exercise could be a promising candidate for enhancing muscle strength in older adults by targeting both muscle atrophy and brain alterations. The increase of muscle strength of elderly is of major concern in order to reduce the risk of falls, improve patients’ quality of life, and potentially reduce mortality risk in certain pathologies (Swallow et al., 2007).

## 5 Methodologials considerations

Several limitations of this study should be acknowledged. First, only quadriceps EMG activity was recorded, without monitoring antagonist muscles such as the hamstrings. This limits our ability to evaluate potential co-contraction strategies, which may influence both movement execution and cortical motor engagement.

Second, although EEG provides high temporal resolution and is well suited for analyzing preparatory cortical dynamics, its spatial resolution remains limited. As a result, deeper or more distributed brain activations, particularly in subcortical or associative regions, may not have been fully captured by our analyses.

Third, due to the structure of the experimental task, participants were required to immediately return their leg to the starting position after each contraction. This continuous movement introduced artifacts in the post-movement period, preventing reliable analysis of termination-related potentials or post-movement cortical dynamics, which may have offered additional insight into inhibitory processes.

Fourth, although a threshold of at least 10 clean trials per condition was applied, the total number of usable trials remained relatively modest for some participants. This could affect the signal-to-noise ratio and reduce the robustness of individual-level averages, particularly in older adults where signal quality is often more variable.

Another limitation is that the older adult group in our study consisted of functionally healthy individuals without clinically significant muscle weakness. While this allowed us to isolate age-related cortical responses under controlled conditions, it limits the generalizability of our findings to frailer or sarcopenic populations. Future studies should examine whether similar cortical responsiveness to eccentric contractions is preserved—or perhaps enhanced—in clinical populations, and whether chronic eccentric training can promote neuroplastic adaptations relevant for rehabilitation.

Finally, all contractions were performed using the right dominant leg. Consequently, the findings may not generalize to the non-dominant limb or to bilateral or functional movements involving interlimb coordination.

## 6 Conclusion

To conclude, this study is the first one to investigate cortical activity during lower limb eccentric contractions in the elderly. Despite an overall reduction in cortical activity in older adults, eccentric contractions were associated with significantly higher cortical activation compared to concentric ones, as in younger individuals. Interestingly, this greater activation is more localized to brain regions associated with motor control of the lower limbs. These findings enhance our understanding of the neural mechanisms underlying muscle contractions in the elderly, providing valuable insights for developing targeted interventions to improve motor function, prevent falls and increase quadriceps muscle strength in this population.

## Data Availability

The raw data supporting the conclusions of this article will be made available by the authors, without undue reservation.
